# Engaging communities in planning and delivering eye care services

**Published:** 2022-09-20

**Authors:** GVS Murthy, BR Shamanna

**Affiliations:** Director: Indian Institute of Public Health, Hyderabad, India and Professor, London School of Hygiene and Tropical Medicine.; Professor: School of Medical Sciences, University of Hyderabad, India.

**Eye care services are successful when they meet the needs of the community, are easy and convenient to access, and have the desired outcomes. This article shows how involving the community in the planning and delivery of eye care services can help to achieve this.**

**Figure:**
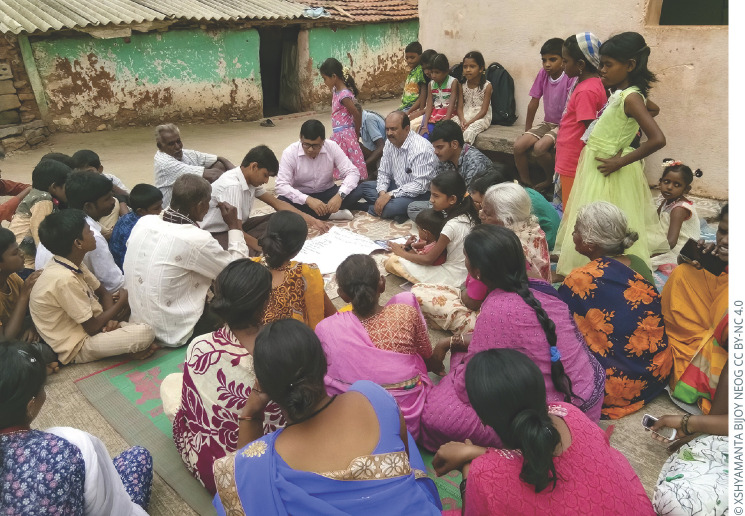
Participatory approach to community eye health activity in Jigani, Karnataka. india

The WHO defines community engagement as “a process of developing relationships that enable stakeholders [e.g., eye care providers and the community] to work together to address health-related issues and promote well-being to achieve positive health impact and outcomes.”^1^

There cannot be a uniform approach to community engagement in the planning of eye care services, as there are socio-cultural differences across countries. However, there are some common principles that apply in all settings, such as fairness, open communication, and transparency. For example, if a community is asked to contribute to the cost of building an eye centre, it would only be fair that they help to decide at what times the centre should be open (e.g., after normal working hours, so that workers can attend appointments) and what level of fees would be sustainable or affordable for them. If this is communicated clearly from the start, and eye care providers provide regular updates and a forum where community members can ask questions, such a venture will be more likely to succeed.

Another benefit of involving communities when planning eye care services is the improved uptake of such services. This is because community engagement can enable changes in behaviour and practices within communities, resulting in better uptake, acceptance, and use of health care services.

According to the Ottawa Charter for Health Promotion,^2^ community engagement requires:

- developing the personal skills of people in the community so they can prepare themselves to cope with a health problem
- strengthening community action by empowering communities to decide for themselves what is best for them and what the benefits of community ownership and control of services are
- creating supportive environments by sustaining natural resources and encouraging a work-leisure balance to improve health,
- building healthy public policy, which includes legislation, taxation, health system changes, etc.
- re-orienting health systems, with more emphasis on disease prevention and health promotion, rather than only concentrating on curative services.

Community engagement and health education leading to eye health-seeking behaviour is something that is central to integrated, people-centred eye care, which is proposed as the way to achieve universal access to eye health care in the World Report on Vision.^3^

Community engagement is a dynamic process. It can be conceptualised as incremental steps of evolution, from minimal engagement to optimal engagement. It means that, over time, health providers (such as hospitals, clinics, or non-governmental organisations) and communities work together to slowly move in the direction of substantial ownership and control of eye care services by the community – such as a vision centre established and managed by a community. Figure 1, adapted proposes a model of different stages of engagement in eye care, (see Figure 1). This has been adapted from an illustration of the degrees of community participation in malaria.^4^

## Examples

### Engaging with communities when planning services

Operation Eyesight Universal supported the vision centre-based community eye health project model in India in 2018. It was designed to promote health-seeking behaviour in communities served by its partner eye hospitals. In this model, vision centres were established as an interface between the target communities and the partner hospitals. Their purpose was to provide underserved and marginalised communities with access to affordable eye care on a sustainable basis. Planning the project involved two steps, namely:

1. Engaging community members by assessing their access to eye care services, preferred eye health providers, the number of people who are visually impaired in the villages, and so on. This was known as a ‘participatory approach to community eye health (PACEH)’.
2. Carrying out a structured knowledge, attitude and practice (KAP) survey amongst community members to identify any knowledge gaps or behaviours that negatively impacted eye health, and then developing and implementing an appropriate and targeted behaviour change communication strategy to address these.

**Figure 1:**
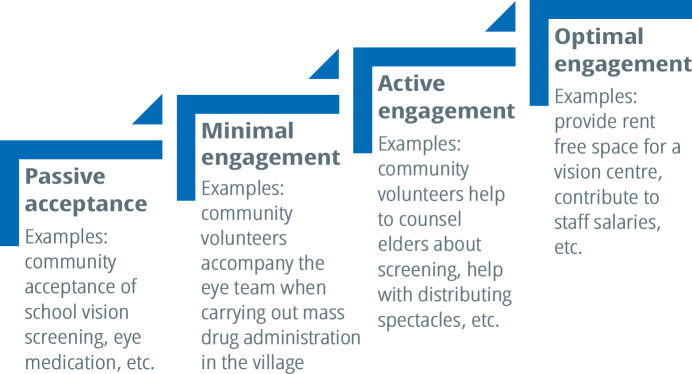
A model of different stages of engagement in eye care

### Engaging with communities to improve uptake

Where fear of surgery is found to be one of the main barriers to communities seeking eye care, inviting a patient advocate or satisfied service user (someone who has undergone successful surgery) to motivate others is a time-tested method that members has been documented over the last four decades.^5^ The eye care team can support patient advocates by providing a set of messages, such as how long surgery takes, how it is done, and when people can return to productive work. It is important to recognise the work of patient coordinators publicly, and – where possible – give them feedback about how many of the people they referred came forward for surgery. This improves their engagement and motivation.

### Involving communities in eye care delivery

The LVPEI Eye Health pyramid model^6^ has, at the base of the pyramid, the Vision Health Guardians (VHGs), who serve a population of 5,000 people each. VHGs are drawn from the community they serve and are either volunteers or receive a small honorarium. They are trained locally for 2 weeks in all aspects of primary eye care and some aspects of primary health care. The main task of a VHG is to create community awareness, conduct school and community screening, distribute spectacles, screen for diabetes and hypertension, and work in coordination with other types of community health workers. A similar system, known as “Friends of the Eye – Nyateros” is also operational in the Gambia.

“There is no such thing as a one-size-fits all approach to community engagement.”

## Conclusion

Community outreach programmes in eye health build on community engagement as an important cog in the wheel of planning, delivery and becoming sustainable.

Although many innovative approaches have been tried, the long-term sustainability of an innovative idea requires careful thought and planning. However, a new idea takes time to mature and show results, and therefore both communities and providers should have the patience to wait, and work for, success – rather than abandoning excellent ideas and innovations prematurely. There is no such thing as a one-size-fits all approach to community engagement, and therefore countries and hospitals need to try out ideas which are relevant in their context.
